# Risk factors associated with non-communicable diseases among government employees in Nepal: insights from a cross-sectional study

**DOI:** 10.3389/fpubh.2025.1514807

**Published:** 2025-05-09

**Authors:** Durga Datta Chapagain, Kennedy Mensah Osei, Danik Iga Prasiska, Heejin Kimm, Vasuki Rajaguru, Sunjoo Kang, Tae Hyun Kim, Sang Gyu Lee, Whiejong Han

**Affiliations:** ^1^Global Health Security, Graduate School of Public Health, Yonsei University, Seoul, Republic of Korea; ^2^Ministry of Health, Hetauda, Bagamati Province, Nepal; ^3^Institute for Health Promotion, Graduate School of Public Health, Yonsei University, Seoul, Republic of Korea; ^4^Department of Healthcare Management, Graduate School of Public Health, Yonsei University, Seoul, Republic of Korea; ^5^Department of Global Health and Disease Control, Graduate School of Public Health, Yonsei University, Seoul, Republic of Korea; ^6^Department of Preventive Medicine, College of Medicine, Yonsei University, Seoul, Republic of Korea

**Keywords:** hypertension, prediabetes, diabetes, non-communicable diseases, associated factors, government employees, Nepal

## Abstract

**Introduction:**

Sedentary lifestyles, unhealthy work environments and occupational stress increase the risk of Non-Communicable Diseases (NCDs) among government employees, impacting healthcare costs and productivity. This study aimed to investigate the prevalence of hypertension, prediabetes, and diabetes, and identify risk factors among government employees in Nepal.

**Methods:**

A cross-sectional study was conducted among 994 government employees. Data on sociodemographic, anthropometric/biochemical measurements, behavioral and clinical factors were collected. Descriptive analysis analyzed the prevalence of NCDs among covariates. Multivariate logistic regression and ROC curves assessed the association between NCDs and covariates/risk factors. Significance was set at *p* < 0.05 and 95% CI.

**Results:**

Participants’ mean age was 33.1 ± 9.1 years, with 82.1% (*n* = 796) male, mostly from aged 30–39 (*n* = 397, 41%), and Brahmin/Chhetri (*n* = 454, 46.9%). Elders had a 6-times higher risk of hypertension (OR: 6.08, CI: 3.1–11.92), above 7-times higher risk of prediabetes (OR: 7.83, CI: 3.32–18.47), and above 16 times higher risk of diabetes (OR: 16.62, CI: 2.5–106.49) compared to aged 18–29. Smoking increased diabetes-risk (OR: 6.82 CI: 1.95–23.8), while alcohol-consumption increased risk of hypertension (OR: 1.51, CI: 1.02–1.63) and prediabetes (OR: 1.88, CI: 1.08–3.28). Overweight increased hypertension risk (OR: 2.79, CI: 1.90–4.09), while obesity increased both hypertension (OR: 3.04, CI: 1.73–5.34) and prediabetes-risk (OR: 2.43, CI: 1.18–4.99).

**Conclusion:**

This study recommends concerned authorities to implement workplace policies for health promotion, intensify awareness campaigns, establish routine screening for government employees, and focus on reducing risk factors and encouraging healthier lifestyles to enhance NCDs prevention.

## Introduction

Non-communicable diseases (NCDs) are a leading cause of death and hospital stays and constitute a major global health burden (1). NCDs account for 41 million annual deaths worldwide, accounting for 74% of all fatalities (1). Of the 17 million NCD-related fatalities that occur annually before the age of 70, low- and middle-income nations account for 86% of these premature deaths and 77% of all NCD deaths (1). The World Health Organization’s (WHO) Southeast Asia Regional Office estimates that NCDs, which include diabetes, cancer, cardiovascular disease, and chronic respiratory illnesses, have a significant and growing negative impact on Southeast Asian health and development. Approximately 9 million deaths, or 62% of all deaths in this region are associated with NCDs (2).

The WHO’s NCDs Progress Monitor 2022 estimates that 117,300 individuals perished in Nepal from NCDs, which accounts for 66% of the total number of deaths (3). In Nepal, the risk of premature death from NCDs is 22% (3). Obesity, increased blood glucose, high Blood Pressure (BP), high cholesterol levels, physical inactivity, alcohol consumption, and smoking are the major risk factors for NCDs (4–10). Prior Studies conducted in globally (11), neighboring country India (12, 13) and Nepal (14) revealed the effect of smoking and alcohol consumption on prevalence of NCDs. Nepal has a particularly high prevalence of certain NCD risk factors, such as smoking, low fruit and vegetable consumption, increased blood pressure, and abnormal lipid levels (9). In addition, there is wide variation in prevalence by age group, sex, place of residence, and ecological zone (15).

Government employees are a key demographic group whose quality of life, health literacy, and behaviors significantly impact the prevention and management of NCDs, as well as broader goals like reducing healthcare costs and improving productivity. However, they face growing vulnerability to NCDs due to sedentary lifestyles, unhealthy work environments, and occupational stress. While studies have highlighted high NCD risk factors such as 99.6% prevalence among metropolitan employees (16), cases of hypertension among hospital health workers (17) and obesity in civil servants of the Ministry of Education and Sports (18), they are often limited to single institutions or narrow geographical regions (16, 19–21), lacking a comprehensive assessment of diverse risk factors across a broader, more heterogeneous government workforce.

To the best of our knowledge, this study constitutes the first comprehensive, large-scale cross-sectional investigation of NCD risk factors among government employees across 50 district-level government offices in Nepal. This research makes a significant scientific contribution by systematically identifying and quantifying context-specific risk factors within a population that has received limited attention in the existing studies. By addressing this critical knowledge gap, this study provides robust, evidence-based insights into the prevalence and determinants of NCDs among government employees. The findings carry important implications for the formulation of targeted workplace health policies and interventions aimed at mitigating NCD risks, thereby promoting improved health outcomes and enhanced productivity within this essential segment of the workforce.

This study aimed to assess the prevalence of non-communicable diseases (NCDs), specifically prediabetes, diabetes, and hypertension; and their associated risk factors and explore the association between these risk factors and NCD occurrence among government employees in the district level government offices at Makwanpur district of Nepal.

## Methods and materials

### Study population and data collection

A cross-sectional study design was used, and the data were collected via a structured survey questionnaire among all government employees working at district-level government offices in the Makwanpur district of Nepal. All the employees were included irrespective of their age, gender, ethnicity, job responsibilities and/or other comorbidities. Lists of the employees were obtained from the concerned government offices. Retired employees or other people who were not enrolled as government employees were excluded. A total of 1,162 eligible individuals from selected government offices were invited to voluntarily participate in the study. Of these, 994 participants (85.5%) provided informed written consent and enrolled in the study. Participation was entirely voluntary, and confidentiality was strictly maintained throughout the process. Comprehensive data was successfully collected across all study variables for these 994 participants. Data analysis was performed on a final sample of 969 participants, following the exclusion of individuals with non-responses to specific variables, including ethnicity (*n* = 1), family history of NCD (*n* = 20), currently smoking (*n* = 1), currently drinking alcohol (*n* = 1), perceived stress (*n* = 3), and BMI (*n* = 1).

The data collection format was developed based on the WHO ‘STEPwise’ Approach to NCD Risk Factor Surveillance (STEPS), last updated on 26 January 2017 (22). The questionnaire incorporated carefully selected items from the STEPS instrument, which were adapted as needed to align with the specific objectives of the study and the local context. The complete data collection tool is provided in the Supplementary materials.

Data collection teams were formed comprising nurses, paramedics, laboratory technicians, and supervisors, including district-level supervisors from the Provincial Health Office and ward in-charges from Hetauda Provincial Hospital. All data collectors and supervisors received 2 days of training on standardized data collection procedures. A pretest was conducted with 5% of the sample size, involving health workers from Hetauda Provincial Hospital and the Provincial Health Office, to evaluate the applicability and clarity of the data collection instruments. Feedback from the pretest was incorporated to refine and improve the final tool. Data obtained during the pretest was not included in the final analysis.

Following training, data collectors coordinated with district offices to schedule data collection, which took place from May to July 2023. Daily supervision ensured completeness and consistency of the collected data.

### Variables

The dependent variables included hypertension and diabetes. Hypertension was defined as systolic blood pressure ≥140 mmHg and/or diastolic ≥90 mmHg, following WHO guidelines (23). Diabetes was identified based on fasting plasma glucose ≥126 mg/dL or postprandial random glucose ≥200 mg/dL, while prediabetes was defined as fasting glucose 100–125 mg/dL or postprandial glucose 140–199 mg/dL, per American Diabetes Association criteria (24).

Independent variables encompassed sociodemographic, behavioral, and anthropometric factors. Sociodemographic data (age, sex, ethnicity, family history of NCDs) and behavioral factors (smoking, alcohol consumption, physical activity, perceived stress) were collected via face-to-face interviews. Age was categorized into four groups (18–29, 30–39, 40–49, 50+). Ethnicity followed the Nepal Demographic and Health Survey 2022 classification (25); which includes six groups: Dalit, Janajati, Madhesi, Muslim, Brahmin/Chhetri, and Others. However, due to the small number of participants in some groups (e.g., Dalit = 2, Muslim = 11, Others = 4), these groups were merged with Madhesi, which had the lowest count among the remaining groups, to form the “Others” category for statistical validity. Insufficient physical activity was defined as engaging in less than 150 min of moderate-intensity exercise or less than 75 min of vigorous-intensity exercise weekly. Currently smoking is defined as having smoked at least once in the past week, while currently drinking alcohol is defined as having consumed alcohol at least once in the past month.

**Figure 1:**
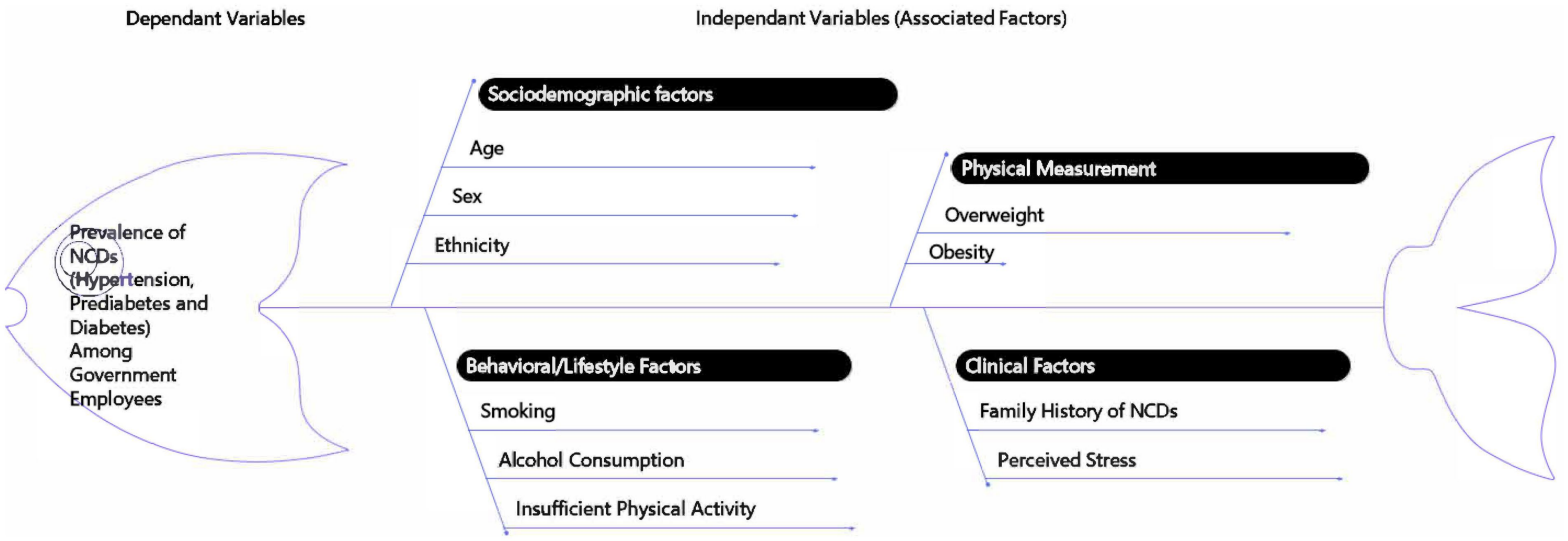
Conceptual framework illustrating the factors associated with non-communicable diseases (NCDs) among government employees in Nepal. The fishbone diagram outlines the dependent variable (prevalence of hypertension, prediabetes, and diabetes) and its association with various covariates and risk factors, including sociodemographic factors, physical measurements, behavioral/lifestyle factors, and clinical factors.

Anthropometric measurements included weight (electronic scale) and height (metal measuring tape), with BMI calculated as weight (kg) divided by height squared (m^2^). BMI was classified per WHO standards: underweight (<18), normal (18–24.9), overweight (25–29.9), and obese (≥30), as no separate national guideline exists in Nepal. The conceptual research design is illustrated in Figure 1.

### Statistical analysis

The data were analyzed using IBM SPSS Statistics 27. Descriptive statistics are presented as frequencies and percentages for all categorical variables. The prevalence of NCDs, including hypertension, prediabetes, and diabetes, with covariates was calculated. The chi-square test was used to assess the significance, as it is a widely used non-parametric method for evaluating relationship between categorical variables (26). Binary logistic regression analysis was utilized to explore the associations of risk factors with hypertension. This method was particularly appropriate because the dependent variable hypertension status is binary. Logistic regression not only evaluates the relationship between predictor variables and the outcome but also estimates odds ratios, providing quantifiable measures of the strength and direction of these associations. For the analysis of risk factors associated with prediabetes and diabetes, we employed multinomial logistic regression, as the dependent variable (diabetes status) included three distinct categories: normal, prediabetes, and diabetes. This analytical method extends the principles of binary logistic regression to accommodate a multi-categorical dependent variable, allowing for a comprehensive assessment of risk factors across multiple outcome levels (27).

In logistic regression, the following categories were taken as the reference groups: age group 18–29, female, Brahmin/Chhetri ethnic group, no history of NCDs, non-currently smokers, non-currently alcohol drinkers, individuals with no physical exercise, no perceived stress, and those classified as normal/underweight according to BMI.

Receiver operating characteristic (ROC) curve analysis was performed for the two continuous predictor variables, age and BMI, to evaluate their predictive capabilities for hypertension, prediabetes, and diabetes. ROC analysis is particularly valuable in assessing the diagnostic accuracy of continuous variables by illustrating the trade-offs between sensitivity and specificity, thus providing a comprehensive understanding of their effectiveness in distinguishing between different disease states (28). *p* < 0.05 was considered to indicate statistical significance for all analyses.

### Ethical considerations

Ethical approval for this research was obtained from the Nepal Health Research Council (NHRC), Government of Nepal (Ref No: 1581, dated 25th March 2024). Additionally, a formal letter of permission (Ref No: 320/2080/81, dated 26th December 2023) for data collection and usage was received from the Health Office Makwanpur under the Bagamati province government. Participants were informed about the study’s objectives, procedures, confidentiality of their information, and their right to withdraw or stop the interview at any time. Informed consent forms were prepared in both Nepali and English languages for the participants’ convenience. Written informed consent was obtained from each participant before data collection commenced. Confidentiality was maintained throughout the study via anonymous data collection. The scanned copies of informed consent forms in both Nepali and English language are included in Supplementary files.

## Results

### Sociodemographic characteristics

Table 1 presents the sociodemographic characteristics of the participants. The mean age of the participants was 33.1 ± 9.1 years, with the largest proportion aged 30–39 years (*n* = 397, 41%), followed by those aged 18–29 years (*n* = 376, 38.8%), indicating that younger adults comprised most respondents. A substantial majority were male (*n* = 796, 82.1%), and nearly half identified as Brahmin/Chhetri (*n* = 454, 46.9%), followed by Janajati (315, 32.5%), reflecting the gender and ethnic distribution within the government workforce. Regarding familial predisposition, 11% reported a family history of hypertension, 7.4% of diabetes, and 3.6% of both, offering insights into potential hereditary risk factors. In terms of behavioral risk factors, 22.1% (*n* = 214) of participants were current smokers, and 31.4% (*n* = 304) reported current alcohol consumption. The majority (*n* = 891, 92%) engaged in vigorous or moderate physical activity, while 27.6% (*n* = 267) reported experiencing stress.

**Table 1:** Socio-demographic characteristics of participants by outcomes (*N* = 969).

Variables	Variables	Total	Hypertension *N* (%)			Diabetes status *N* (%)			
		*N* (%)	No	Yes	*p*-value	None	Prediabetes	Diabetes	*p*-value
Age in years (mean ± SD = 33.1 ± 9.1)	18–29	376 (38.8)	345 (91.8)	31 (8.2)	***	360 (95.7)	14 (3.7)	2 (0.5)	***
	30–39	397 (41)	309 (77.8)	88 (22.2)		360 (90.7)	34 (8.6)	3 (0.8)	
	40–49	134 (13.8)	86 (64.2)	48 (35.8)		109 (81.3)	19 (14.2)	6 (4.5)	
	50 and above	62 (6.4)	36 (58.1)	26 (41.9)		41 (66.1)	16 (25.8)	5 (8.1)	
Sex	Female	173 (17.9)	156 (90.2)	17 (9.8)	***	160 (92.5)	12 (6.9)	1 (0.6)	0.318
	Male	796 (82.1)	620 (77.9)	176 (22.1)		710 (89.2)	71 (8.9)	15 (1.9)	
Ethnicity	Brahmin/Chhetri	454 (46.9)	365 (80.4)	89 (19.6)	0.679	403 (88.8)	41 (9)	10 (2.2)	0.346
	Janajati	315 (32.5)	255 (81)	60 (19)		291 (92.4)	21 (6.7)	3 (1)	
	Others	200 (20.6)	156 (78)	44 (22)		176 (88)	21 (10.5)	3 (1.5)	
Family history of NCD	None	755 (77.9)	618 (81.9)	137 (18.1)	0.079	690 (91.4)	56 (7.4)	9 (1.2)	***
	Hypertension	107 (11)	76 (71)	31 (29)		96 (89.7)	9 (8.4)	2 (1.9)	
	Diabetes	72 (7.4)	57 (79.2)	15 (20.8)		59 (81.9)	9 (12.5)	4 (5.6)	
	Both hypertension and diabetes	35 (3.6)	25 (71.4)	10 (28.6)		25 (71.4)	9 (25.7)	1 (2.9)	
Currently smoking	No	755 (77.9)	622 (82.4)	133 (17.6)	***	689 (91.3)	60 (7.9)	6 (0.8)	***
	Yes	214 (22.1)	154 (72)	60 (28)		181 (84.6)	23 (10.7)	10 (4.7)	
Currently alcohol consumption	No	665 (68.6)	561 (84.4)	104 (15.6)	***	612 (92)	45 (6.8)	8 (1.2)	**
	Yes	304 (31.4)	215 (70.7)	89 (29.3)		258 (84.9)	38 (12.5)	8 (2.6)	
Physical exercise	No	78 (8)	59 (75.6)	19 (24.4)	0.306	59 (75.6)	17 (21.8)	2 (2.6)	***
	Yes	891 (92)	717 (80.5)	174 (19.5)		811 (91)	66 (7.4)	14 (1.6)	
Perceived stress	No	702 (72.4)	566 (80.6)	136 (19.4)	0.492	641 (91.3)	52 (7.4)	9 (1.3)	**
	Yes	267 (27.6)	210 (78.7)	57 (21.3)		229 (85.8)	31 (11.6)	7 (2.6)	
BMI classification	Normal/Underweight	548 (56.6)	490 (89.4)	58 (10.6)	***	512 (93.4)	30 (5.5)	6 (1.1)	***
	Overweight	336 (34.7)	231 (68.8)	105 (31.3)		291 (86.6)	36 (10.7)	9 (2.7)	
	Obese	85 (8.8)	55 (64.7)	30 (35.3)		67 (78.8)	17 (20)	1 (1.2)	

*p*-value * = <0.05, ** = <0.01 and *** = <0.001.

With respect to body weight, above one third (34.7%, *n* = 336) of participants were classified as overweight and 8.8% (*n* = 85) as obese, highlighting a significant burden of weight-related risk factors among the study population.

### Prevalence of NCDs

The prevalence of all three NCDs among respondents with various risk factors and covariates is shown in Table 1. Individuals aged 50 years and above had the highest prevalence of hypertension, prediabetes, and diabetes (41.9, 25.8, and 8.1%, respectively), indicating that older adults are more susceptible to NCDs. Hypertension was more prevalent among men (22.1%) than women (9.8%). In contrast, no significant association was observed between sex and the prevalence of prediabetes or diabetes. The prevalence of the three non-communicable diseases (NCDs) did not differ significantly among the various ethnic groups, suggesting an absence of major racial disparities in disease distribution.

Diabetes was more prevalent (5.6%) among individuals with a family history of diabetes, while prediabetes was more common (25.7%) among those with a family history of both illnesses, hypertension and diabetes. In contrast, no significant association was found between the prevalence of these NCDs and a family history of hypertension. The prevalence of prediabetes (10.7%), diabetes (4.7%), and hypertension (28%) was greater among current smokers compared to non-smokers. The results showed that those who were currently consuming alcohol had greater rates of diabetes (2.6%), hypertension (29.3%), and prediabetes (12.5%) than those who were not. The prevalence of prediabetes (21.8%) and diabetes (2.6%) was higher among individuals with insufficient physical exercise. Similarly, participants experiencing any form of stress exhibited an increased prevalence of prediabetes (11.6%) and diabetes (2.6%). However, neither physical exercise nor perceived stress showed a statistically significant association with hypertension prevalence.

BMI was found to be statistically significant with the prevalence of NCDs. Obese people had the highest prevalence of hypertension and prediabetes (35.3 and 20% respectively), followed by overweight (30.8 and 10.7% respectively). The prevalence of diabetes was highest (2.6%) among overweight participants. These results underscore the significant impact of body weight on the risk of NCDs. All the above values were found to be statistically significant.

### Associations between NCDs and covariates

Table 2 illustrates the results of binary and multinomial logistic regression analyses carried out to determine the associations between NCDs and covariates. The study reveals that respondents aged over 50 years were substantially more likely to develop hypertension, prediabetes, and diabetes compared to younger age groups (18–29 years). Older adults had a 6-fold higher likelihood of developing hypertension (OR: 6.08, CI: 3.1–11.92), above 7-fold increase in odds to develop prediabetes (OR: 7.83, CI: 3.32–18.47) and a staggering above 16-fold higher likelihood of developing diabetes (OR: 16.62, CI: 2.5–106.49) than those aged 18–29 years. The data demonstrate that aging significantly increases the risk of chronic conditions, with men showing higher odds of hypertension than women (OR: 2.45, CI: 1.33–4.50). Ethnicity was not significantly associated with NCDs, suggesting that lifestyle and sociodemographic factors may be more influential. A family history of diabetes strongly predicted the condition diabetes (OR: 9.8, CI: 2.22–43.20), though it was not associated to hypertension. Prediabetes risk was notably higher in those with a family history of both diabetes and hypertension (OR: 4.46, CI: 1.83–10.90), indicating combined genetic and environmental influence. Current smokers had over six odds of diabetes (OR: 6.82, CI: 1.95–23.8), while alcohol consumption was associated with increased odds of hypertension (OR: 1.51, CI: 1.02–2.23) and prediabetes (OR: 1.88, CI: 1.08–3.28), though not with diabetes. Physical exercise was protective against prediabetes (OR: 0.21, CI: 0.1–0.41) and hypertension (OR: 0.52, CI: 0.28–0.97), but not significantly associated with diabetes. Perceived stress showed no significant association with NCDs. Obesity significantly increased the risk of hypertension (OR: 3.04, CI: 1.73–5.34) and prediabetes (OR: 2.43, CI: 1.18–4.99), while overweight individuals also had elevated hypertension risk (OR: 2.79); however, BMI was not associated with diabetes.

**Table 2:** Association of NCDs (hypertension, prediabetes, and diabetes) with the covariates/risk factors (*N* = 969).

Variables	Covariates	Hypertension		Prediabetes		Diabetes	
		OR (95% CI)	*p*-value	OR (95% CI)	*p*-value	OR (95% CI)	*p*-value
Age (Years)	18–29	Ref.		Ref.		Ref.	
	30–39	2.19 (1.37–3.51)	***	1.77 (0.89–3.52)	0.105	1.01 (0.15–6.87)	0.990
	40–49	4.09 (2.36–7.08)	***	3.35 (1.52–7.36)	**	9.26 (1.52–56.37)	*
	50 and above	6.08 (3.10–11.92)	***	7.83 (3.32–18.47)	***	16.62 (2.50–106.49)	**
Sex	Female	Ref.	**	Ref.	0.191	Ref.	0.252
	Male	2.45 (1.33–4.50)		1.69 (0.77–3.70)		3.92 (0.38–40.71)	
Ethnicity	Brahmin/Chhetri	Ref.		Ref.		Ref.	
	Janajati	1.02 (0.68–1.52)	0.938	0.75 (0.42–1.36)	0.351	0.64 (0.15–2.66)	0.537
	Others	1.23 (0.78–1.93)	0.378	1.58 (0.86–2.91)	0.143	0.84 (0.18–3.82)	0.822
Family history of NCD	No History	Ref.		Ref.		Ref.	
	Hypertension	1.65 (0.99–2.74)	0.054	1.10 (0.50–2.42)	0.806	1.84 (0.34–10.03)	0.481
	Diabetes	1.01 (0.52–1.97)	0.972	1.47 (0.63–3.44)	0.376	9.80 (2.22–43.20)	**
	Both hypertension and diabetes	1.46 (0.64–3.33)	0.365	4.46 (1.83–10.90)	***	3.64 (0.38–34.60)	0.261
Currently smoking	No	Ref.	0.760	Ref.	0.794	Ref.	**
	Yes	1.07 (0.70–1.63)		0.92 (0.50–1.69)		6.82 (1.95–23.80)	
Currently drinking alcohol	No	Ref.	*	Ref.	*	Ref.	0.620
	Yes	1.51 (1.02–2.23)		1.88 (1.08–3.28)		0.73 (0.22–2.49)	
Physical exercise	No	Ref.	*	Ref.	***	Ref.	0.142
	Yes	0.52 (0.28–0.97)		0.21 (0.10–0.41)		0.27 (0.05–1.56)	
Perceived stress	No	Ref.	0.894	Ref.	0.232	Ref.	0.367
	Yes	0.97 (0.66–1.43)		1.37 (0.82–2.29)		1.69 (0.54–5.31)	
BMI classification	Normal/underweight	Ref.		Ref.		Ref.	
	Overweight	2.79 (1.90–4.09)	***	1.54 (0.89–2.67)	0.122	1.73 (0.54–5.54)	0.357
	Obese	3.04 (1.73–5.34)	***	2.43 (1.18–4.99)	*	0.54 (0.06–5.08)	0.594

*p*-value * = <0.05, ** = <0.01 and *** = <0.001; Ref., Reference category.

### Subgroup analysis of receiver operating characteristic (ROC) curves

**Figure 2:**
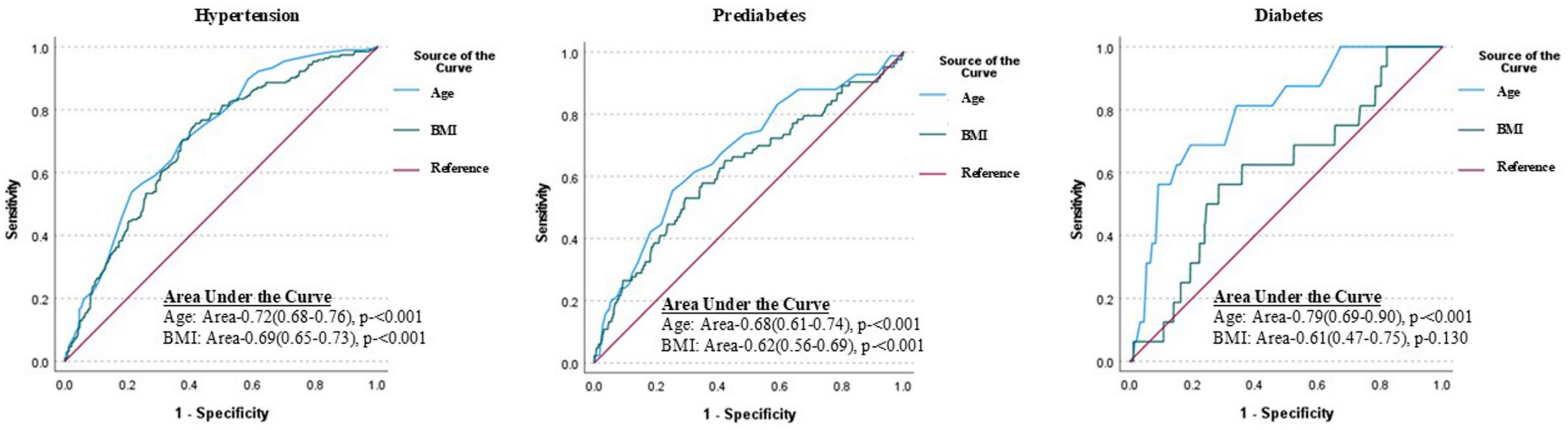
Receiver operating characteristic (ROC) curves demonstrating the predictive performance of age and BMI for hypertension, prediabetes, and diabetes among the government employees in Nepal. Age shows better discriminative ability than BMI for all conditions, with the strongest performance for diabetes (AUC = 0.79, *p* < 0.001). While BMI also displays significance for hypertension and prediabetes (*p* < 0.001) it is not significant for diabetes prediction (*p* = 0.13).

Figure 2 illustrates the predictive ability of age and BMI for identifying the risk of hypertension, prediabetes, and diabetes. Age consistently demonstrated a stronger predictive ability compared to BMI across all conditions. For hypertension, age yielded an Area Under the Curve (AUC) of 0.72, indicating good predictive accuracy, while BMI had moderate AUC of 0.69; both associations were statistically significant (*p* < 0.001). Similarly, for prediabetes, age showed moderate predictive ability with an AUC of 0.68, compared to BMI with an AUC of 0.62, and both were significant (*p* < 0.001).

Notably, the predictive performance of age was most pronounced for diabetes, achieving the highest AUC of 0.79, reflecting strong diagnostic accuracy (*p* < 0.001). In contrast, BMI’s predictive ability for diabetes was weaker, with an AUC of 0.61, and this association was not statistically significant (*p* = 0.130). These findings emphasize the prominent role of age in predicting these non-communicable diseases (NCDs), particularly diabetes, compared to BMI. This analysis underscores the differential contributions of age and BMI in disease prediction and their potential implications for risk stratification and targeted interventions.

## Discussion

This cross-sectional study evaluated the prevalence of risk factors and three NCDs: diabetes, prediabetes, and hypertension. The study also investigated the associations between these risk factors and NCDs among government employees. The findings highlight a complex interaction between demographic, hereditary, and lifestyle factors in the prevalence of hypertension, prediabetes, and diabetes. They emphasize the need for targeted interventions focusing on modifiable risk factors such as smoking, alcohol use, physical inactivity, and poor weight management to effectively reduce the burden of non-communicable diseases in the population. The results of our research indicated that older adults among government employees are more likely to have prediabetes, diabetes, and hypertension (above 7-fold, above 16-fold and 16-fold higher than younger adults respectively), compared to previous findings. The highest risk group for all three NCDs were participants 50 years of age and above. A study in China (29) revealed elders had more than double risk of prediabetes and about 50% higher risk of diabetes compared to middle aged in general population. Similar result showed by the studies in Nepal (9, 10). A study conducted among health workers in the central hospital in Nepal provided the similar results for hypertension prevalence in elders as double risk compared to middle aged and 6 times higher compared to younger adults (17). Although, the PEN Package is launched by the Ministry of Health and Population in collaboration with WHO, which aims to strengthen the detection and management of NCDs at the primary healthcare level. The aging population and rising NCD burden require the nation to prioritize NCD prevention and control. Furthermore, we discovered that men had over double prevalence of hypertension, which is consistent with other studies’ findings (9, 15). The presence of both diabetes and hypertension in the family increased the risk of prediabetes, whereas a family history of diabetes increased the chance of acquiring diabetes, in line with other research findings (30–32). However, the research did not find a statistically significant association between a family history of hypertension with any of the three NCDs that were investigated.

The prevalence of current smoking among government employees was 22.1%, closely aligning with the nationwide survey by the Nepal Health Research Council in 2019 (22.2%) for the general population. However, our study reveals a higher prevalence of current alcohol consumption among government employees (31.4%) compared to the national average (24.6%). Similarly, a study conducted in Nepal in general population (9) and in health workers (17) identified employed individuals had a greater prevalence of alcohol consumption than did the general population. These findings underscore the need for targeted awareness programs to help reduce risk factors including alcohol consumption and smoking to prevent NCDs in this workforce. The possibility of having diabetes was found to be significantly greater among current smokers. Nicotine increases blood sugar levels and is more difficult to control. Smokers with diabetes frequently require higher insulin dosages to maintain blood sugar levels that are near the target (33). Another study in Iran revealed that the incidence of diabetes types 1 and 2 increased with smoking habits (34). According to the American College of Cardiology (48), smoking, particularly in younger adults, may exacerbate hypertension. Even when taking blood pressure medication, smokers have difficulty controlling their blood pressure. However, we could not find an association between hypertension and smoking. A Mendelian randomization meta-analysis conducted in Britain also suggested that smoking is causally related to a higher resting heart rate but not to alterations in blood pressure or the risk of hypertension (35).

We found alcohol consumption increased the risk of developing hypertension and prediabetes by 51 and 88% among government employees. Results of a previous study also showed greater effect of alcohol consumption as it increased the 4.5 times higher risk of hypertension in health workers in Nepal (17). Similarly, alcohol consumption was found to be associated with elevated blood pressure, increasing DBP by 7.5 (95% CI: 3.7–11.3) mm Hg and SBP by 9.5 (95% CI: 3.8–15.1) mm Hg, according to a study performed in Southeast China (36). However, we could not find the relationship between alcohol consumption and diabetes in our study. In contrast, several previous studies revealed a nonlinear relationship between alcohol consumption and the risk of diabetes in which low and moderate alcohol consumption were associated with a lower risk of diabetes, with the risk of diabetes increasing above the threshold (37, 38).

In our study, 8% of employees were found to be physically inactive, which is slightly higher than the result (7.4%) reported in the 2019 STEPS survey of the general population (30). A high level of physical activity was shown to protect against the development of prediabetes, diabetes, and hypertension, consistent with the findings of numerous prior studies (5, 6, 39–42). Strategies that target the biological risk factors related to physical activity are essential in this specific population to halt the rising occurrence of NCDs. Potential workplace policies and interventions are required, such as promoting ergonomic workstations, introducing structured physical activity programs, and addressing workplace stress through wellness initiatives. Our findings revealed a greater percentage of overweight and obese government workers (34.7 and 8.8%, respectively) in line with a study conducted in civil servants in Nepal as 33.4% (18) compared to a study conducted in general population by the NHRC, MoHP, Nepal in 2019 (23.5 and 7.2%, respectively) (39). BMI was found to be associated with the prevalence of prediabetes and hypertension but not to that of diabetes. By comparing overweight and obese people to normal weight people, we found that their risk of developing hypertension is about 3 times greater, which aligns about 4.5 times higher in the result of previous study in civil servants in Nepal (18). A few earlier investigations conducted in the USA (43, 44) produced findings similar to these results. Numerous studies conducted in both developed and developing nations have shown an association between obesity and overweight with prediabetes as well as diabetes (44–46). The American Heart Association also proposed obesity and weight management as a standard of care for type 2 diabetes in 2023 (47). Similarly, in our study, obesity was shown to be associated with prediabetes, while overweight was not found to be significantly associated with prediabetes, and BMI was not substantially associated with diabetes.

Findings from the ROC analysis have notable implications for health policy and workplace wellness programs tailored to government employees. Given the strong association of age with these NCDs, targeted screening and early intervention strategies should prioritize age-specific risk assessments. Such initiatives could include mandatory periodic health check-ups focused on early detection of NCDs among aging employees, coupled with age-adjusted preventive measures and counseling. While BMI showed relevance in predicting hypertension and prediabetes, workplace programs should also address lifestyle modifications, such as promoting physical activity, balanced nutrition, and stress management, to mitigate the risks associated with these conditions.

### Limitations

Several confounding variables, such as marital status, education level, socio-economic status, dietary habits, and diversity of jobs such as type of job (physical work or table work), and duration of working hours might have influenced the results. However, due to time constraints, minimizing disruption to participants’ working hours and limited resources, these factors could not be accounted for in this study. Future research should aim to incorporate these variables to enable a more comprehensive understanding of risk factors for non-communicable diseases among government employees.

Some information such as drinking habits, smoking habits, physical activity, and feelings of stress, relies on self-reported data, which carries a risk of bias, such as underreporting due to fear of criticism. To address this, data collectors were trained to ensure consistency and neutrality in their approach, minimizing interviewer bias. A pretest involving 5% of the sample was conducted to refine data collection tools. Emphasis on anonymity and confidentiality encouraged truthful responses, while triangulation, through cross-verification with available records, enhanced the accuracy of the data.

Although the WHO BMI classification standards are widely used and adopted in Nepal, we recognize their potential limitations in accurately reflecting the unique genetic, cultural, and body composition differences within the Nepalese population.

As this study was limited to government employees within a single district of Nepal, its findings may not be broadly applicable to individuals in other professions, regions, or international settings due to potential variations in workplace conditions, cultural norms, and access to healthcare services. Cross-sectional studies, by design, are limited in establishing causal relationships between risk factors and NCDs.

The higher representation of male participants aligns with the gender distribution in the government employees, where males are numerically dominant. Consequently, this study may not fully capture gender-specific differences in non-communicable disease (NCD) risk factors and experiences, potentially limiting the generalizability of findings across genders.

## Conclusion and implications

This study highlighted a significant prevalence of risk factors for NCDs among government employees, placing them at greater risk for diabetes and hypertension. The findings emphasize the need for targeted interventions to address excessive alcohol use, physical inactivity, and obesity in all age groups, with an emphasis on older adults. Surveillance systems tailored to government employees should be established to monitor and mitigate these risks effectively.

### Policy recommendations

To support the health and well-being of government employees in Nepal, we recommend workplace policies that promote physical activity through wellness programs, address stress via mental health initiatives, and reduce sedentary behavior through ergonomic interventions. Additional measures include regular health screening, encouraging physical activity through facilities or incentives, awareness campaigns to limit alcohol and tobacco use, and fostering healthy dietary choices in workplace canteens. Integrating these strategies into national NCD prevention frameworks can improve employee well-being, productivity, and satisfaction while setting a model for workplace health initiatives across Nepal. The government of Nepal could play a pivotal role in fostering these changes by integrating workplace health into national NCD prevention strategies. By prioritizing the health and well-being of employees, such policies would not only reduce NCD risk factors but also contribute to improved productivity and overall workplace satisfaction.

### Future research directions

Further studies could adopt an in-depth qualitative approach to explore the lived experiences and perceptions of government employees regarding their health behaviors and challenges. Understanding their perspectives could uncover barriers to healthier lifestyles and provide insights into cultural or workplace-specific influences on health outcomes. Additionally, future research could include broader cohorts of government employees and address variables not covered in this study such as marital status, education level, socio-economic status, dietary habits, and diversity of jobs such as type of job (physical work or table work), and duration of working hours.

## Data Availability

The raw data supporting the conclusions of this article will be made available by the authors, without undue reservation.

## Supplementary material

SUPPLEMENTARY FILE 1 Information collection instrument.

SUPPLEMENTARY FILE 2 Informed consent form in English and Nepali language.

## References

[1] World Health Organization. (2023). Non-communicable diseases. Available online at: https://www.who.int/news-room/fact-sheets/detail/noncommunicable-diseases (Assessed November 20, 2023).

[2] World Health Organization. (2023). SEARO. Available online at: https://www.who.int/southeastasia/health-topics/noncommunicable-diseases (Assessed November 24, 2023).

[3] World Health Organization. (2022). Noncommunicable diseases progress monitor 2022. Available online at: https://www.who.int/publications/i/item/9789240047761 (Assessed October 10, 2023).

[4] Krishnamoorthy Y Nagarajan R Murali S. Effectiveness of multiple combined lifestyle interventions in reducing blood pressure among patients with prehypertension and hypertension: a network meta-analysis. J Public Health. (2023) 45:E319–31. doi: 10.1093/pubmed/fdac027, PMID: 35211753

[5] Lemes ÍR Sui X Turi-Lynch BC Lee DC Blair SN Fernandes RA. Sedentary behaviour is associated with diabetes mellitus in adults: findings of a cross-sectional analysis from the Brazilian National Health System. J Public Health. (2019) 41:742–9. doi: 10.1093/pubmed/fdy169, PMID: 30260410

[6] Christofaro DGD Ritti-Dias RM Chiolero A Fernandes RA Casonatto J de Oliveira AR. Physical activity is inversely associated with high blood pressure independently of overweight in Brazilian adolescents. Scand J Med Sci Sports. (2013) 23:317–22. doi: 10.1111/j.1600-0838.2011.01382.x, PMID: 22092334

[7] Kuruvilla A Mishra S Ghosh K. Prevalence and risk factors associated with non-communicable diseases among employees in a university setting: a cross-sectional study. Clin Epidemiol Glob Health. (2023) 21:101282. doi: 10.1016/j.cegh.2023.101282, PMID: 40266665

[8] Florence GE Derman WE Popperwell JM Kunorozva L Gomez-Ezeiza J. Prevalence of health risk behaviours related to non-communicable diseases amongst south African university students: a systematic review. J Public Health. (2023) 45:1042–55. doi: 10.1093/pubmed/fdad106, PMID: 37409582 PMC10688999

[9] Bista B Dhimal M Bhattarai S Neupane T Xu YY Pandey AR. Prevalence of non-communicable diseases risk factors and their determinants: Results from STEPS survey 2019, Nepal. PLoS One. (2021) 16:e0253605. doi: 10.1371/journal.pone.0253605, PMID: 34329300 PMC8323895

[10] Sapkota BP Baral KP Rehfuess EA Parhofer KG Berger U. Effects of age on non-communicable disease risk factors among Nepalese adults. PLoS One. (2023) 18:e0281028. doi: 10.1371/journal.pone.0281028, PMID: 37267282 PMC10237426

[11] Bai J Shi F Ma Y Yang D Yu C Cao J. The global burden of type 2 diabetes attributable to tobacco: a secondary analysis from the global burden of disease study 2019. Front Endocrinol. (2022) 13:905367. doi: 10.3389/fendo.2022.905367, PMID: 35937829 PMC9355706

[12] Adhikari K Gupta N. Tobacco use: a major risk factor for non-communicable diseases in Central Nepal. Res J Pharm Biol Chem Sci. (2014) 5:19–24.

[13] Mishra VK Srivastava S Muhammad T Murthy PV. Relationship between tobacco use, alcohol consumption and non-communicable diseases among women in India: evidence from National Family Health Survey-2015-16. BMC Public Health. (2022) 22:713. doi: 10.1186/s12889-022-13191-z, PMID: 35410193 PMC8996590

[14] Sharma SR Matheson A Lambrick D Faulkner J Lounsbury DW Vaidya A. The role of tobacco and alcohol use in the interaction of social determinants of non-communicable diseases in Nepal: a systems perspective. BMC Public Health. (2020) 20:1368. doi: 10.1186/s12889-020-09446-2, PMID: 32894104 PMC7487957

[15] Aryal KK Mehata S Neupane S Vaidya A Dhimal M Dhakal P. The burden and determinants of non-communicable diseases risk factors in Nepal: findings from a nationwide STEPS survey. PLoS One. (2015) 10:e0134834. doi: 10.1371/journal.pone.0134834, PMID: 26244512 PMC4526223

[16] Neupane R Bhandari TR. Prevalence of non-communicable diseases and its associate factors among government employees in Biratnagar, Nepal. JNMA J Nepal Med Assoc. (2018) 56:497–503. doi: 10.31729/jnma.3476, PMID: 30058632 PMC8997319

[17] Ghimire P Khadka A Anuwatnonthakate A Trongsakul S. Prevalence and factors associated with hypertension among health workers of central hospitals in Nepal. Indones J Public Health. (2020) 15:325–38. doi: 10.20473/ijph.v15i3.2020.325-338

[18] Simkhada P Poobalan A Simkhada PP Amalraj R Aucott L. Knowledge, attitude, and prevalence of overweight and obesity among civil servants in Nepal. Asia Pac J Public Health. (2011) 23:507–17. doi: 10.1177/1010539509348662, PMID: 19825841

[19] Mishra SR Neupane D Shakya A Adhikari S Kallestrup P. Modifiable risk factors for major non-communicable diseases among medical students in Nepal. J Community Health. (2015) 40:863–8. doi: 10.1007/s10900-015-0012-6, PMID: 25833419

[20] Timalsina P Singh R. Assessment of risk factors of non-communicable diseases among Semiurban population of Kavre District, Nepal. J Environ Public Health. (2021) 2021:1–7. doi: 10.1155/2021/5584561, PMID: 34211559 PMC8205567

[21] Schlabach A Guragain B Marx B Espesete D Shirilla B Warbrick J. Non-communicable disease risk factors and prevalence within Thaha, Makwanpur, Nepal: a cross-sectional study. J Glob Health Rep. (2021) 5:244. doi: 10.29392/001c.22244

[22] World Health Organization. WHO STEPS Surveillance Manual. Geneva: World Health Organization (2017).

[23] World Health Organization. (2023). First WHO report details devastating impact of hypertension and ways to stop it. Available online at: https://www.who.int/news/item/19-09-2023-first-who-report-details-devastating-impact-of-hypertension-and-ways-to-stop-it (Assessed December 11, 2023).

[24] Diagnosis_American Diabetes Association. (2023). Understanding diabetes diagnosis. available online at: https://diabetes.org/about-diabetes/diagnosis (Assessed December 20, 2023).

[25] Shah R. (2023). Nepal demographic and health survey 2022 Ministry of Health and population new ERA Ministry of Health and population. Available online at: https://www.dhsprogram.com/ (Assessed December 24, 2023).

[26] Das BK Jha DN Sahu SK Yadav AK Raman RK Kartikeyan M. Chi-Square test of significance In: Das BK Jha DN Sahu SK Yadav AK Raman RK Kartikeyan M, editors. Concept building in fisheries data analysis. Singapore: Springer (2023). 81–94.

[27] Gail M Krickeberg K Samet JM Tsiatis A Wong W. Statistics for biology and health series. New York: Springer (2007).

[28] Fawcett T. An introduction to ROC analysis. Pattern Recogn Lett. (2006) 27:861–74. doi: 10.1016/j.patrec.2005.10.010

[29] Yan Z Cai M Han X Chen Q Lu H. The interaction between age and risk factors for diabetes and prediabetes: a community-based cross-sectional study. Diabetes Metab Syndr Obes. (2023) 16:85–93. doi: 10.2147/DMSO.S390857, PMID: 36760587 PMC9843502

[30] Alijanvand MH Aminorroaya A Kazemi I Amini M Yamini SA Mansourian M. Prevalence and predictors of prediabetes and its coexistence with high blood pressure in first-degree relatives of patients with type 2 diabetes: a 9-year cohort study. J Res Med Sci. (2020) 25:31. doi: 10.4103/jrms.JRMS_472_18, PMID: 32419788 PMC7213002

[31] Wagner R Thorand B Osterhoff MA Müller G Böhm A Meisinger C. Family history of diabetes is associated with higher risk for prediabetes: a multicentre analysis from the German Center for Diabetes Research. Diabetologia. (2013) 56:2176–80. doi: 10.1007/s00125-013-3002-1, PMID: 23979484

[32] Ranasinghe P Cooray DN Jayawardena R Katulanda P. The influence of family history of hypertension on disease prevalence and associated metabolic risk factors among Sri Lankan adults chronic disease epidemiology. BMC Public Health. (2015) 15:576. doi: 10.1186/s12889-015-1927-7, PMID: 26092387 PMC4475303

[33] CDC. Centers for Disease Control and Prevention. Georgia: CDC (2024).

[34] Molla GJ Ismail-Beigi F Larijani B Khaloo P Moosaie F Alemi H. Smoking and diabetes control in adults with type 1 and type 2 diabetes: a Nationwide study from the 2018 National Program for prevention and control of diabetes of Iran. Can J Diabetes. (2020) 44:246–52. doi: 10.1016/j.jcjd.2019.07.002, PMID: 31494031

[35] Linneberg A Jacobsen RK Skaaby T Taylor AE Fluharty ME Jeppesen JL. Effect of smoking on blood pressure and resting heart rate: a Mendelian randomization meta-analysis in the CARTA consortium. Circ Cardiovasc Genet. (2015) 8:832–41. doi: 10.1161/CIRCGENETICS.115.001225, PMID: 26538566 PMC4684098

[36] Zhao PP Xu LW Sun T Wu YY Zhu XW Zhang B. Relationship between alcohol use, blood pressure and hypertension. J Epidemiol Community Health. (1979) 73:796–801. doi: 10.1136/jech-2018-211185, PMID: 31227586

[37] Knott C Bell S Britton A. Alcohol consumption and the risk of type 2 diabetes: a systematic review and dose-response meta-analysis of more than 1.9 million individuals from 38 observational studies. Diabetes Care. (2015) 38:1804–12. doi: 10.2337/dc15-0710, PMID: 26294775

[38] Holst C Becker U Jørgensen ME Grønbæk M Tolstrup JS. Alcohol drinking patterns and risk of diabetes: a cohort study of 70,551 men and women from the general Danish population. Diabetologia. (2017) 60:1941–50. doi: 10.1007/s00125-017-4359-3, PMID: 28748324

[39] Pazmino L Esparza W Ramón Aladro-Gonzalvo A León E. Impact of work and recreational physical activity on prediabetes condition among U.S. adults: NHANES 2015-2016. Int J Environ Res Public Health. (2021) 18:1378. doi: 10.3390/ijerph 33546150 PMC7913268

[40] Smith AD Crippa A Woodcock J Brage S. Physical activity and incident type 2 diabetes mellitus: a systematic review and dose–response meta-analysis of prospective cohort studies. Diabetologia. (2016) 59:2527–45. doi: 10.1007/s00125-016-4079-0 27747395 PMC6207340

[41] Færch K Witte DR Brunner EJ Kivimäki M Tabák A Jørgensen ME. Physical activity and improvement of glycemia in prediabetes by different diagnostic criteria. J Clin Endocrinol Metab. (2017) 102:3712–21. doi: 10.1210/jc.2017-00990, PMID: 28973497 PMC5630255

[42] Lee JY Ryu S Sung KC. Association of baseline level of physical activity and its temporal changes with incident hypertension and diabetes mellitus. Eur J Prev Cardiol. (2018) 25:1065–73. doi: 10.1177/2047487318774419, PMID: 29719968

[43] Bramlage P Pittrow D Wittchen HU Kirch W Boehler S Lehnert H. Hypertension in overweight and obese primary care patients is highly prevalent and poorly controlled. Am J Hypertens. (2004) 17:904–10. doi: 10.1016/j.amjhyper.2004.05.017, PMID: 15485752

[44] Hall ME Cohen JB Ard JD Egan BM Hall JE Lavie CJ. Weight-loss strategies for prevention and treatment of hypertension: a scientific statement from the American Heart Association. Hypertension. (2021) 78:E38–50. doi: 10.1161/HYP.0000000000000202, PMID: 34538096

[45] Khamseh ME Sepanlou SG Hashemi-Madani N Joukar F Mehrparvar AH Faramarzi E. Nationwide prevalence of diabetes and prediabetes and associated risk factors among Iranian adults: analysis of data from PERSIAN cohort study. Diabetes Ther. (2021) 12:2921–38. doi: 10.1007/s13300-021-01152-5, PMID: 34595726 PMC8521563

[46] Regmi D Al-Shamsi S Govender RD Al KJ. Incidence and risk factors of type 2 diabetes mellitus in an overweight and obese population: a long-term retrospective cohort study from a gulf state. BMJ Open. (2020) 10:e035813. doi: 10.1136/bmjopen-2019-035813, PMID: 32616491 PMC7333876

[47] Elsayed NA Aleppo G Aroda VR Bannuru RR Brown FM Bruemmer D. Obesity and weight management for the prevention and treatment of type 2 diabetes: standards of Care in Diabetes—2023. Diabetes Care. (2023) 46:S128–39. doi: 10.2337/dc23-S008, PMID: 36507637 PMC9810466

[48] American College of Cardiology. (2016). Heart Disease and Stroke Statistics: 2016 Update Available at: https://www.acc.org/latest-in-cardiology/ten-points-to-remember/2016/01/05/13/08/heart-disease-and-stroke-statistics-2016-update (Assessed December 22, 2023).

