# Supplementary material for: Risk factors associated with non-communicable diseases among government employees in Nepal: insights from a cross-sectional study
Source: Front Public Health. 2025 May 9;13:1514807. doi: 10.3389/fpubh.2025.1514807 (PMC12098267; doi:10.3389/fpubh.2025.1514807)
Supplement: SUPPLEMENTARY FILE 1 — Information collection instrument. [file Data_Sheet_1.pdf]

Supplementary file 1: Information Collection Instrument

Research Title: Risk Factors Associated with Non-Communicable Diseases Among Government Employees in Nepal: Insights from a Cross-Sectional Study

Data Collection Questionnaire

1. Survey Information		
**Questions**	**Response**	**Code**
Employee Office/workplace ID	|__|__|__|__|__|__|	**I1**
Employee Office/workplace name<br>*Enter Office/workplace name as appropriate.*		**I2**
Interviewer ID<br>*Enter interviewer's identification.*	|__|__|__|	**I3**
Date of completion of the instrument<br>*Enter date when instrument completed.*	|__|__| |__|__| |__|__|__|__| dd mm year	**I4**
Consent has been read and obtained<br>*Select relevant response.*	Yes 1<br>No 2 **If NO, END**	**I5**
Interview Language<br>*Select relevant response.*	English 1<br>Nepali 2	**I6**
Time of interview<br>(24-hour clock)<br>*Enter time interview started.*	|__|__| : |__|__| hrs mins	**I7**
**Additional Information that may be helpful**		
Family Surname (Optional)<br>*Enter family surname (reassure the participant on the confidential nature of this information and that this is only needed for follow up).*		**I8**
First Name (Optional)<br>*Enter first name of respondent (re-assure the participant of the confidential nature of this information and that this is only needed for follow up).*		**I9**
Contact phone number where possible (Optional)<br>*Enter phone number (reassure the participant on the confidential nature of this information and that this is only needed for follow up).*		**I10**
**2. Demographic Information**		
Sex (*Record Male / Female as observed)*<br>*Select Male / Female as observed.*	Male 1<br>Female 2	**D1**
How old are you?<br>*If the age is unknown, help participant estimate their age by interviewing them about their recollection of widely known major events.*	Years |__|__|	**D2**
What is your *ethnic* **background**?<br>*Select the relevant ethnic group to which the participant belongs.*	Dalit 1<br>Janajati 2<br>Madhesi 3<br>Muslim 4<br>Brahmin/ Chhetri 5<br>Others 6<br>Refused 88	**D3**

3. Behavioral Measurements

Do you **currently** smoke any **tobacco** products, such as cigarettes, cigars or pipes (within past week)?<br>*Ask the participant to think of any tobacco products he/she is smoking currently.*	Yes 1<br>No 2	**B1**
Have you consumed any alcohol within the **past 30 days**?<br>*Select the appropriate response. Even if the participant has only consumed a few sips of alcohol in the past 30 days, the response should be "Yes".*	Yes 1<br>No 2	**B2**

Physical Activity

Next, I am going to ask you about the time you spend doing different types of physical activity in a typical week. Please answer these questions even if you do not consider yourself to be a physically active person.

Think first about the time you spend doing work. Think of work as the things that you have to do such as paid or unpaid work, study/training, household chores, harvesting food/crops, fishing or hunting for food, seeking employment or any vigorous-intensity sports, fitness or recreational (leisure) activities that cause large increases in breathing or heart rate like [running or football]. In answering the following questions 'vigorous-intensity activities' are activities that require hard physical effort and cause large increases in breathing or heart rate, 'moderate-intensity activities' are activities that require moderate physical effort and cause small increases in breathing or heart rate.

Read this opening statement out loud. It should not be omitted. The respondent will have to think first about the time he/she spends doing work (paid or unpaid work, household chores, harvesting food, fishing or hunting for food, seeking employment, then about the time he/she travels from place to place, and finally about the time spent in vigorous as well as moderate physical activity during leisure time.

Remind the respondent when he/she answers the following questions that 'vigorous-intensity activities' are activities that require hard physical effort and cause large increases in breathing or heart rate, 'moderate-intensity activities' are activities that require moderate physical effort and cause small increases in breathing or heart rate. The participants should only consider those activities undertaken continuously.

Do you involve in vigorous-intensity activities that cause large increases in breathing or heart rate like *[carrying or lifting heavy loads, digging or construction work] any vigorous-intensity sports, fitness or recreational (leisure) activities that cause large increases in breathing or heart rate like [running or football] for at least 75 minutes per week*?<br>*Ask the participant to think about vigorous-intensity activities at work/travel or sports, fitness or recreational activities. Activities are regarded as vigorous intensity if they cause large increases in breathing and/or heart rate.*	Yes 1<br>No 2	**B3**
Do you involved in moderate-intensity activities that cause small increases in breathing or heart rate such as brisk walking [or carrying light loads] any moderate-intensity sports, fitness or recreational (leisure) activities that cause a small increase in breathing or heart rate such as brisk walking, [cycling, swimming, volleyball] for at least 150 minutes per week?<br>*Ask the participant to think about moderate-intensity activities at work/travel or sports, fitness or recreational activities. Activities are regarded as moderate intensity if they cause small increases in breathing and/or heart rate.*	Yes 1<br>No 2	**B4**

4. Medical Information

Perceived Stress

Have you experienced any form of stress recently, such as workplace-related stress, severe-financial stress, stressful life-events, or other types of stress impacting your well-being?	Yes 1<br>No 2	**M1**
Are you currently taking any medication or undergoing any form of treatment to manage stress, such as therapy, counselling, or other stress-management interventions recommended by a healthcare professional?	Yes 1<br>No 2	**M2**

Family History of NCDs

Has any member of your family ever been diagnosed with **raised blood pressure or hypertension** by a licensed doctor or certified health worker, or have they received treatment for hypertension prescribed or administered by an authorized healthcare professional?	Yes 1<br>No 2	**FH1**
Has any member of your family ever been diagnosed with **raised blood glucose level or diabetes** by a licensed doctor or certified health worker, or have they received treatment for hypertension prescribed or administered by an authorized healthcare professional?	Yes 1<br>No 2	**FH2**

5. Physical Measurements

**Blood Pressure**		
Interviewer ID<br>*Record interviewer ID (in most cases interviewer would be the same as for behavioural measurements).*	└─┴─┴─┘	**BP1**
Device ID for blood pressure<br>*Record device ID.*	└─┴─┘	**BP2**
Reading 1<br>*Record first measurement after the participant has rested for 15 minutes. Wait 3 minutes before taking second measurement.*	Systolic (mmHg) └─┴─┴─┘	**BP3a**
	Diastolic (mmHg) └─┴─┴─┘	**BP3b**
Reading 2<br>*Record second measurement. Ask the participant to rest for another 3 minutes before taking the third measurement.*	Systolic (mmHg) └─┴─┴─┘	**BP4a**
	Diastolic (mmHg) └─┴─┴─┘	**BP4b**
Reading 3<br>*Record third measurement.*	Systolic (mmHg) └─┴─┴─┘	**BP5a**
	Diastolic (mmHg) └─┴─┴─┘	**BP5b**
Average of three readings<br>*Calculate average of the three readings*	Systolic (mmHg) └─┴─┴─┘	**BP6a**
	Diastolic (mmHg) └─┴─┴─┘	**BP6b**
During the past two weeks, have you been treated for raised blood pressure with drugs (medication) prescribed by a doctor or other health worker?<br>*Select an appropriate response.*	Yes 1<br>No 2	**BP7**
**BMI**		
**For women:** Are you pregnant?<br>*Pregnant women skip over height, weight measurements.*	Yes 1 If *Yes, go to G1*<br>No 2	**BM1**
Interviewer ID<br>*Record interviewer ID (in most cases interviewer would be the same as for behavioural and blood pressure measurements).*	└─┴─┴─┘	**BM2**
Device IDs for height and weight<br>*Record device IDs.*	Height └─┴─┘<br>Weight └─┴─┘	**BM3a**<br>**BM3b**
Height<br>*Record participant's height in meter with one decimal point.*	in Meters (m) └─┴─┴─┘ . └─┘	**BM4**
Weight<br>*Record participant's weight in kg with one decimal point.*	in Kilograms (kg) └─┴─┴─┘ . └─┘	**BM5**
Body Mass Index (BMI) (*one decimal point*)<br>*Calculate BMI using the formula = weight(kg)/[height(m)]²*	└─┴─┴─┘ . └─┘ kg/m²	**BM6**

6. Biochemical Measurements

**Blood Glucose**		
During the past 12 hours have you had anything to eat or drink, other than water?<br>*It is essential that the participant has fasted.*	Yes 1<br>No 2	**G1**
Technician ID<br>*Record ID of the person taking the measurement.*	└─┴─┴─┘	**G2**
Device ID (*Record device ID).*	└─┴─┘	**G3**
Time of day blood specimen taken (24-hour clock)<br>*Enter time measurement started.*	Hours: minutes └─┴─┘ : └─┴─┘	**G4**
Fasting blood glucose (*Double check that the participant has fasted)*	mg/dl └─┴─┘ . └─┴─┘	**G5**
Random blood glucose<br>*If the participant has eaten or drink, other than water*	mg/dl └─┴─┘ . └─┴─┘	**G6**
Today, have you taken insulin or other drugs (medication) that have been prescribed by a doctor or other health worker for raised blood glucose?	Yes 1<br>No 2	**G7**

Thank you for your participation

Supplementary file. 2: Informed Consent Form in English and Nepali Language

Risk Factors Associated with Non-Communicable Diseases Among Government Employees in Nepal: Insights from a Cross-Sectional Study

Informed Consent Form for the Participants (English)

Statement

We are conducting research entitled "Risk Factors Associated with Non-Communicable Diseases Among Government Employees in Nepal: Insights from a Cross-Sectional Study". We have come with you to collect the necessary information for this research.

Purpose of this research

To investigate the burden and association of risk factors with 3 major NCDs (hypertension, pre-diabetes and diabetes) among government employees working in Makwanpur district of Nepal.

Methods

We will ask about age, sex, race, family history of non-communicable diseases, history of treatment, and behavioral and lifestyle information like alcohol and tobacco use, physical activity, and perceived stress. We will measure your height, weight, and blood pressure, among other basic parameters. To check your blood sugar level, we will take a tiny sample of blood from your fingertip. After that, the data will be analyzed to find the prevalence of non-communicable diseases and the degree of association with their risk factors.

Expected duration of the participation and frequency of contact

- The interview, measurements, and blood sample collection could take a total of 20 minutes.
- To collect information, participants only need to make one contact.

Benefits

Although participants won't receive any immediate financial benefits, they will be able to find out quickly about their blood pressure, body mass index, and blood sugar levels.

Risks

Participants won't be at any risk, although the process of drawing blood from your fingertip may be uncomfortable due to minor pain.

Payment/reimbursement

During the data collection process, participants do not need to pay cost associated with blood pressure checks, BMI assessments, or blood sugar tests. Participants will not receive any payment, compensation, free treatment, reimbursement for incidental costs, or insurance coverage if diseases are identified during a physical and laboratory examination.

Voluntary participation/withdrawal

Your participation in the study is entirely voluntary, and you are free to refuse to participate at any time, withdraw your consent at any time, and refuse to answer any interview questions that you do not want to answer.

Study team

- **Principal investigator**: Durga Datta Chapagain
- **Co-investigators:** Danik Iga Prasiska, Osei Mensah Kennedy, Prof. Heejin Kimm, Prof. Vasuki Rajaguru, Prof. Sun Joo Kang, Prof. Whiejong Han

Contact details

You can contact at the following address if you have any queries related to the study and/or helpline for appeal against violation of ethical principles and human rights:

1. Principal investigator: Durga Datta Chapagain
Ph.D. Student, Yonsei University, South Korea
Cellphone no: 01046385507 (South Korea)
9851201026 (Nepal: WhatsApp)
Email: ddchapagain@gmail.com,
Email: dchapagain@yonsei.ac.kr

2, Ethical Review M&E Section
Nepal Health Research Council (NHRC)
Ramshah path, Kathmandu
Post Office Box 7626
Kathmandu, Nepal Fax: 977-1-4262469 / 4268284
Tel.: +977 - 4254220 (Ext no 125)
E-mail: approval@nhrc.gov.np,
E-mail: ethicalreviewb@gmail.com,
URL: https://erb.nhrc.gov.np

Use of data

This data will be used for analysis of the burden of non-communicable diseases and associated factors among government employees and publication of the findings in a journal. Your participation and the data provided will be completely confidential. Your personal details will not be shared in any report of the study.

The results of this survey will be shared to the concerned government authorities which will help plan strategies in reducing the risk factors that contribute to non-communicable diseases in Nepal.

After reading or listening to the details mentioned above, we are hoping that you will participate in this study. Are you willing to participate in this study?

Yes ☐ **No** ☐

Name of the participant:

Signature

Date:

“Risk Factors Associated with Non-Communicable Diseases Among Government Employees in Nepal: Insights from a Cross-Sectional Study”

विषयमा अध्ययन गर्न

Informed Consent Form for the Participants (Nepali)
सहभागीहरूको लागि सूचित सहमति फारम (नेपाली)

कथन: हामी नेपालमा सरकारी कर्मचारीहरूमा नसर्ने रोगसँग सम्बन्धित कारकहरू सम्बन्धि अनुसन्धान गर्दैछौं | यसको लागि आवश्यक विवरण संकलन गर्न हजुरहरु समक्ष आएका छौं |

उद्देश्य: यस अनुसन्धानको उद्देश्य नेपालको मकवानपुर जिल्लामा कार्यरत सरकारी कर्मचारीहरूका तीन प्रमुख नसर्ने रोगहरु (उच्च रक्तचाप, प्रि-मधुमेह र मधुमेह), यिनीहरुका जोखिम कारकहरूको बोझ र सम्बद्धताको खोजी गर्ने रहेको छ |

अनुसन्धानका विधिहरु: हामी तपाइको जनसांख्यिकीय जानकारी जस्तै उमेर, लिंग, जाति, नसर्ने रोगहरुको पारिवारिक इतिहास, नसर्ने रोगहरुको उपचारको इतिहास, रक्सी र धुम्रपान प्रयोग सहित व्यवहार र जीवनशैली सम्बन्धि जानकारी, शारीरिक व्यायाम, र तनाव बारे केहि प्रश्नहरु सोध्नेछौं । हामी तपाईंको उचाइ, तौल र रक्तचाप सहित केही सरल मापन गर्नेछौं | हामी तपाईंको रगतमा चिनीको मात्रा जाँच गर्न तपाईंको औंलाको टुप्पोबाट थोरै मात्रामा रगत सङ्कलन गर्नेछौं । यसरी संकलित विवरणहरुको विश्लेषण गरि सरकारी कर्मचारीहरूमा तीन प्रमुख नसर्ने रोगहरु (उच्च रक्तचाप, प्रि-मधुमेह र मधुमेह) को अवस्था र जोखिम कारकहरूको सम्बद्धताको खोजी गर्नेछौं |

सहभागिताको अपेक्षित अवधि र सम्पर्क गर्नुपर्ने पटक: अन्तर्वार्ता, शारीरिक मापन र रगतको नमूना संकलन गर्दा कुल २० मिनेट जति लाग्न सक्छ । जानकारी सङ्कलनको लागि सहभागीहरूसंग केवल एक पटक मात्र भेट गरिने छ ।

विवरण संकलनबाट हुने फाइदाहरू: विवरण उपलब्ध गराएवापत सहभागीहरूले कुनै पनि आर्थिक लाभ तथा उपहारहरू प्राप्त गर्ने छैनन्, तथापी आफ्नो स्वास्थ्य अवस्था (रक्तचाप, BMI, र रगतमा चिनीको स्तर) को बारेमा तत्काल जानकारी प्राप्त गर्न सक्नेछन ।

जोखिमहरू: विवरण संकलनको कारणले सहभागीहरूलाई कुनै पनि जोखिम हुनेछैन, यद्यपि तपाईंको औंलाको टुप्पोबाट रगत निकाल्ने प्रक्रियामा सानो दुखाइको कारणले केहि असहज हुन सक्छ ।

भुक्तानी / क्षतिपूर्ति नहुने: विवरण सङ्कलन प्रक्रियामा सहभागीहरूले रक्तचाप परिक्षण, BMI मूल्याङ्कन, वा रगतमा चिनीको मात्र परिक्षण गरेवापत कुनै पनि शुल्क तिर्नु पर्ने छैन | यदि शारीरिक र प्रयोगशाला परीक्षणको क्रममा कुनै रोगहरू पहिचान भएमा सहभागीहरूले कुनै पनि उपचार भुक्तानी, क्षतिपूर्ति, निःशुल्क उपचार, आकस्मिक लागतको लागि प्रतिपूर्ति, वा बीमा सुबिधा प्राप्त गर्ने छैनन् ।

स्वैच्छिक सहभागिता/ छोड्न पाइने: यो अध्ययनमा तपाईंको सहभागिता पूर्णतया स्वैच्छिक हो, र तपाईं कुनै पनि समयमा भाग लिन अस्वीकार गर्न, कुनै पनि समयमा आफ्नो सहमति फिर्ता लिन, र तपाईंले जवाफ दिन नचाहेको कुनै पनि अन्तर्वार्ता प्रश्नहरूको जवाफ दिन अस्वीकार गर्न स्वतन्त्र हुनुहुन्छ ।

अनुसन्धान टोलीको विवरण

प्रमुख अन्वेषक: दुर्गादत्त चापागाईं, पीएचडी विद्यार्थी, योन्सी विश्वविद्यालय, दक्षिण कोरिया

सह अन्वेषकहरु: डानिक इगा प्रसिस्का, ओसेई मेन्साह केनेडी, प्रो. हिजिन किम, प्रो. वासुकी राजागुरु, प्रो. सन जू कांग, प्रो. व्हिजंग हान हुनुहुन्छ | वहाँहरु सबै योन्सी विश्वविद्यालय, दक्षिण कोरियाबाट हुनुहुन्छ |

सम्पर्क विवरण

यस अनुसन्धानको विवरण संकलनको क्रममा नैतिक सिद्धान्त तथा मानव अधिकारको उल्लङ्घन भएमा अपीलको लागि एवम् अध्ययन र/वा हेल्पलाइनसँग सम्बन्धित कुनै पनि प्रश्नहरूको लागि तपाईंले निम्न ठेगानाहरूमा सम्पर्क गर्न सक्नुहुनेछ:

१. प्रमुख अन्वेषक: दुर्गा दत्त चापागाई

पीएच.डी. विद्यार्थी, योन्सेई विश्वविद्यालय, दक्षिण कोरिया

मोबाइल नम्बर: ०१०४६३८५५०७ (दक्षिण कोरिया); ९८५१२०१०२६ (नेपाल: व्हाट्सएप)

इमेल: ddchapagain@gmail.com, dchapagain@yonsei.ac.kr

२. नैतिक समीक्षा (Ethical Review) अनुगमन तथा मुल्यांकन शाखा

नेपाल स्वास्थ्य अनुसन्धान परिषद् (NHRC)

रामशाह पथ, काठमाडौं, नेपाल, पोस्ट बक्स न. ७६२६

फ्याक्स: ९७७-१-४२६२४६९ / ४२६८२८४

टेलिफोन: +९७७ - ४२५४२२० (विस्तार नम्बर १२५)

इमेल: approval@nhrc.gov.np, ethicalreviewb@gmail.com,

URL: https://erb.nhrc.gov.np

तथ्यांकको प्रयोग: यस तथ्याङ्कलाई सरकारी कर्मचारीमा हुने नसर्ने रोगको बोझ र त्यससँग सम्बन्धित कारकहरूको विश्लेषण र निष्कर्ष निकाल्न तथा जर्नलमा प्रकाशन गर्न प्रयोग गरिनेछ । तपाइँको सहभागिता र प्रदान गरिएको विवरण पूर्ण रूपमा गोप्य रहनेछ । तपाईंको व्यक्तिगत विवरणहरू अध्ययनको कुनै पनि प्रतिवेदन वा प्रकाशनमा उल्लेख गरिने छैन ।

यस सर्वेक्षणको नतिजा सम्बन्धित सरकारी अधिकारीहरूलाई साझा गरिनेछ जसले नेपालमा नसर्ने रोगहरू निम्त्याउने जोखिम कारकहरूलाई कम गर्न रणनीति तथा योजना बनाउन मद्दत गर्नेछ ।

माथिका सम्पूर्ण विवरण अध्ययन वा श्रवण गरेपछि हामी आशा गर्छौं कि तपाईं यस अध्ययनमा सहभागी हुनुहुनेछ। के तपाईं यस अध्ययनमा भाग लिन इच्छुक हुनुहुन्छ ?

इच्छुक छु ☐ **इच्छुक छैन** ☐

सहभागीको पुरा नाम:

हस्ताक्षर:

मिति:
